# Identification and Mapping of a New Soybean Male-Sterile Gene, *mst-M*

**DOI:** 10.3389/fpls.2019.00094

**Published:** 2019-02-06

**Authors:** Qingsong Zhao, Ya Tong, Chunyan Yang, Yongqing Yang, Mengchen Zhang

**Affiliations:** ^1^The Key Laboratory of Crop Genetics and Breeding of Hebei, Institute of Cereal and Oil Crops, Hebei Academy of Agriculture and Forestry Sciences, Shijiazhuang, China; ^2^Root Biology Center, College of Resources and Environment, Fujian Agriculture and Forestry University, Fuzhou, China

**Keywords:** male sterility, abnormal pollen grains, inheritance test, bulked segregant analysis, dCAPS

## Abstract

The use of sterility is common in plants and multiple loci for hybrid sterility have been identified in crops such as rice. In soybean, fine-mapping and research on the molecular mechanism of male sterility is limited. Here, we identified a male-sterile soybean line, which produces larger, abnormal pollen grains that stain poorly with I_2_-KI. In an inheritance test, all *F*_1_ plants were fertile and the *F*_2_ and *F*_2:3_ populations conformed with the expected segregation ratio of 3:1 (fertility:sterility) (*p* = 0.82) and showed a 1:2:0 ratio of homozygous fertile: heterozygous fertile: homozygous sterile genotypes (*p* = 0.73), suggesting that the sterility was controlled by a single recessive gene (designated “*mst-M*”). Bulked segregant analysis showed that almost all single-nucleotide polymorphisms (SNPs; 95.92%) were distributed on chromosome 13 and 868 SNPs (95.81%) were distributed in the physical region of Chromosome 13.21877872 to Chromosome 13.22862641. Genetic mapping revealed that *mst-M* was flanked by *W1* and dCAPS-1 with genetic distances of 0.6 and 1.8 cM, respectively. The order of the consensus markers and known sterility genes was: Satt146 – (5.0 cM) – *st5* – (2.5 cM) – Satt030 – (15.3 cM) – *ms6* – (5.0 cM) – Satt149 – (39.5 cM) – *W1* – (0.6 cM) – *mst-M* – (14.1 cM) – Satt516 (7.5 cM) – *ms1* – (16.3 cM) – Satt595. These results suggest that *mst-M* is a newly identified male-sterility gene, which represents an alternative genetic resource for developing a hybrid seed production system for soybean.

## Introduction

Sterility is a common phenomenon among plants. On the basis of the mode of inheritance, two main types of sterility have been identified in plants: cytoplasmic sterility and nucleus-dependent sterility (Zhang et al., 2008; Chen and Liu, 2014; Yang et al., 2014; Speth et al., 2015; Bohra et al., 2016; Chang et al., 2016; Liu et al., 2018; Xie et al., 2018). In plant breeding, hybrid seeds/lines are advantageous because they produce high yields. In comparison to female sterility, male sterility, including cytoplasmic male sterility (CMS) and genetic male sterility (GMS), has wide applications in commercial crop hybrids because male sterility greatly increases the effectiveness of *F*_1_ hybrid seed production without manual pollination and can dramatically reduce production costs (Cheng et al., 2007; Xu et al., 2007; Chen et al., 2011; Huang et al., 2014). The most successful application of male sterility in crop hybrid seed production is in rice (*Oryza sativa*; Cheng et al., 2007; Huang et al., 2014). In recent years, many male sterility genes have been identified and cloned, and the underlying genetic and molecular mechanisms have been described. For example, *MS1*, a newly evolved gene in wheat (*Triticum aestivum*; Poaceae), is specifically expressed in microsporocytes and is essential for microgametogenesis (Wang et al., 2017). *OsPKS2* encodes a polyketide synthase that is involved in pollen wall formation in rice. Male sterility can be caused by *OsPKS2* mutation (Zou et al., 2018). Two-line hybrid rice was developed based on the discovery of photoperiod-sensitive male sterility (PSMS) germplasm, which is male-sterile under long-day conditions but fertile under short-day conditions (Fan et al., 2016). Recently, the molecular mechanism of a PSMS gene, *Pms1*, which encodes a long-non-coding RNA, PMS1T, was elucidated (Fan et al., 2016). In addition, other male-sterility genes, such as *ZmMs33* in maize (*Zea mays*; Xie et al., 2018) and *MSH1* in *Brassica juncea* (Zhao et al., 2016), have been cloned and well characterized.

Soybean is an economically important crop that is grown worldwide for the high contents of oil (20–25%) and protein (42–45%) in its seeds (Adak and Kibritci, 2016). Although soybean is extremely important for human consumption, it has a lower yield than other important food crops, such as wheat, rice, and maize (Wen et al., 2016; Gao et al., 2018; Ma et al., 2018). Although a three-line system based on CMS has been developed in soybean, large-scale hybrid seed production is difficult to achieve because of the extremely low frequency of natural cross-pollination (Ray et al., 2003). Therefore, the application of male sterility in soybean hybrid seed-production research has typically lagged behind other crops. At least three CMS restorer loci have been identified in soybean (Wang et al., 2016). The CMS restorer locus *Rf-m* was fine-mapped to chromosome 16 between the flanking markers GmSSR1602 and GmSSR1610 with genetic distances of 0.11 and 0.2502 cM, respectively, and a pentatricopeptide repeat gene was predicted to be the candidate restorer gene. Yang et al. (2010) mapped two independent *Rf* loci linked to *Satt626* in molecular linkage group (MLG) M and *Satt300* in MLG A1 at genetic distances of 9.75 and 11.18 cM, respectively. With regard to GMS, more than 20 male-sterility loci have been reported, including *ms1*–*ms9*, *msMOS*, *msp*, *st1*–*st8*, *ASR-7-206*, *A03-2137*, *A05-133*, *A06-204*, and *st_A06-2/6* (Owen, 1928; Hadley and Starnes, 1964; Palmer, 1974, 2000; Palmer et al., 1978, 2008; Delannay and Palmer, 1982; Palmer and Kaul, 1983; Graybosch and Palmer, 1985, 1988; Graybosch et al., 1987; Horner and Palmer, 1995; Jin et al., 1997, 1998; Palmer and Horner, 2000; Cervantes-Martinez et al., 2007, 2009; Rebeccaa et al., 2011; Baumbach et al., 2012), the majority of which have been mapped to a linkage group (Yang et al., 2014; Speth et al., 2015). However, none of these loci have been successfully cloned or fine-mapped. Therefore, the molecular mechanisms of male sterility in soybean are largely unknown.

In this study, we identified a soybean male-sterile mutant line from a soybean breeding line. We investigated the genetic mechanism of the mutant line and fine-mapped the locus for male sterility. Our results provide a foundation for cloning male-sterility genes and elucidating the molecular mechanism of male sterility in soybean.

## Materials and Methods

### Plant Materials

In our soybean breeding program, we observed an advanced-generation soybean breeding line (here designated “Fertility wild type”, “F-wt”) that showed segregation for sterility. The sterile line is here designated “Sterility-Mutant” (St-M). The St-M line was preserved in the heterozygous F-wt/St-M line. Soybean ‘Jidou No. 12’ (JD12) was used as the male or female parent in crosses with St-M. *F*_1_, *F*_2_, *F*_2:3_, BCF_1_, BCF_1:2_, and BCF_2_ populations and a *F*_5:6_ residual heterozygous (RH) line were developed to investigate the genetic mechanism of sterility and fine-map the sterility gene in St-M.

### Morphological Analysis

Anthers from unopened flowers were excised and stained in 1% iodine–potassium iodide (I_2_-KI) solution. The morphology of stained pollen grains, including the shape, size, and color of the grains, was observed under a Leica EZ4 HD microscope. Images of stained pollen grains were captured using a Leica DM2000 LED microscope. The total number of pollen grains of each anther in the visual field was artificially counted, with nine replications for each phenotype.

Mature pollen grains of St-M and F-wt plants were dissected and immediately examined under a scanning electron microscope (SEM) to determine morphological differences between the two lines following Willingham and Rutherford (1984). The diameter of the observed pollen grains was measured according to the scale of the pollen SEM images.

### Statistical Analysis

The segregation of sterility phenotypes was evaluated in the *F*_1_, *F*_2_, *F*_2:3_, BCF_1_, BCF_1:2_, and BCF_2_ populations. Goodness of fit to theoretical ratios was assessed using the chi-square (χ^2^) test in accordance with methods described by Bailey (2012), i.e., χ_c_^2^ = Σ(|*E*_O_ -*E*|- 0.5)^2^/*E* or χ^2^ = Σ(*E*_O_ -*E*)^2^/*E*, where *E*_O_ and *E* are the observed and expected frequencies, respectively. The Yates correction factor (0.5) was applied in the χ_c_^2^ calculation with one degree of freedom (Brown, 2004).

### Development of dCAPS Markers

Primers for the derived cleaved amplified polymorphic sequences used in this study were designed using the dCAPS Finder 2.0 program^1^ (Neff et al., 2002). Because no restriction site adjacent to the mutation site was available, a single nucleotide mismatch was introduced adjacent to the single-nucleotide polymorphism (SNP) position to create a restriction enzyme recognition site in the amplicon of one allele but not the other. The genotypes of the tested soybean plants were determined with the dCAPS marker. The PCR amplification reactions were carried out as follows. The reaction mixture contained 1 μL (50 ng) template DNA, 2.5 μL of 10× Ex Taq^TM^ HS buffer, 2 μL of 0.2 mM dNTPs, 0.125 μL (1 U) Ex Taq HS DNA polymerase, 0.5 μL (15 pmol) of each forward and reverse primer, and water to make up the volume to 25 μL. The following PCR amplification procedure was adjusted according to the primer set used: 95°C for 5 min, and then 30 cycles of 95°C for 30 s, 53–58°C for 30 s, and 72°C for 30 s, followed by a final extension at 72°C for 10 min. The amplicons were digested using suitable restriction endonucleases in a final volume of 25 μL in accordance with the manufacturer’s instructions. The digested products were separated by electrophoresis in a 3% agarose gel and visualized by staining with ethidium bromide.

### DNA Extraction and SSR Marker Analysis

Total genomic DNA was extracted from soybean leaves using the cetyltrimethylammonium bromide (CTAB) method following Doyle (1991) with minor modifications. Sixteen simple sequence repeat (SSR) markers located on soybean chromosome 13 flanking the male sterility gene were selected from the Soybase database^2^. Marker polymorphism between St-M and JD12 was evaluated. Finally, three polymorphic SRR markers, namely Satt516, Satt146, and Satt149, were selected for genotyping of progenies. For SSR analysis, amplification reactions were carried out as described for the dCAPs marker analysis. The PCR products amplified by the SSR primers were visualized after electrophoresis in an 8% polyacrylamide gel followed by silver staining. All primers were synthesized by Shanghai Invitrogen Biotechnology Co., Ltd. (Shanghai, China).

### Bulked Segregant Analysis Based on Genomic DNA Resequencing

Based on the phenotypes of the offspring, a single RH plant segregating for sterility and fertility was identified and selected from the *F*_6_ generation. Seeds from the RH plant were individually harvested to form a sub-*F*_2_ population, which consisted of 135 plants. Equal amounts of leaves from male-sterile *F*_2_ plants were sampled and homogenized, whereas leaf samples from male-fertile *F*_2_ plants were collected individually. The genotypes of the male-fertile *F*_2_ plants were determined based on their progenies. Equal amounts of leaves from homozygous *F*_2_ plants were sampled and homogenized. Finally, two pools were formed from leaves of 34 plants homozygous for male sterility and leaves of 36 plants homozygous for male fertility, respectively. Genomic DNA was extracted from the homogenized sample pools using the established CTAB protocol (Doyle, 1991). The genomic DNA was used to construct sequencing libraries with the TruSeq Library Construction Kit following the manufacturer’s protocol, and the libraries were sequenced using an Illumina Hi-seq platform. The sequencing reads were aligned to the soybean reference genome Williams 82.a2.v1 using the BWA software^3^ with default parameters. Subsequent processing, including removal of duplicate reads, was performed using SAMtools and PICARD^4^. The raw SNP sets were called by SAMtools with the parameters ‘-q 1 -C 50 -m 2 -F 0.002 -d 1000.’ We then filtered the data sets using the following criteria: (1) mapping quality >100; (2) depth of the variate position >1; and (3) homozygous type at the variate position.

### Scoring and Linkage Analysis

The genotype and phenotype for each *F*_2_ plant were recorded based on SSR or dCAPS allelic patterns. A plant was scored as homozygous for the male-sterile parent alleles (A), homozygous for the male-fertile parent alleles (B), or heterozygous for the *F*_1_ alleles (H). An asterisk (^∗^) was used to represent missing data. The phenotype of male sterility and white flowers was coded as “A,” and other phenotypes were coded as “D.” Based on the genotype and phenotype scores, a linkage map was constructed using JoinMap 4.1 with Kosambi mapping methods (Kosambi, 2016). Linkage maps were drawn using the MapChart 2.2 software (Voorrips, 2002).

## Results

### Phenotypic Characterization of Pollen Grains From Sterile and Fertile Plants

**FIGURE 1:**
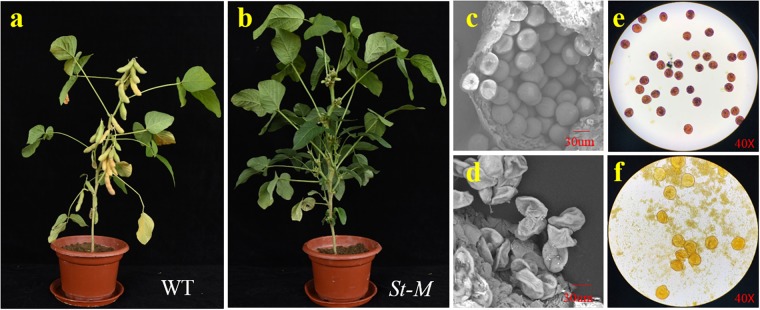
Phenotypic characterization of the soybean sterile line (St-M) and fertile wild type (F-wt). Morphology of **(a)** St-M and **(b)** F-wt plants at the R7 growth stage. Scanning electron micrographs of pollen grains from **(c)** St-M and **(d)** F-wt. Pollen grains stained with I_2_-KI from **(e)** St-M and **(f)** F-wt.

The growth and development of the sterile line (St-M) and the fertile wild-type parent (F-wt) were compared over an entire growing season. No difference in phenotype between St-M and F-wt was observed before flowering. From the R1 growth stage, St-M plants showed early abscission of flowers or development of small, fleshy but seedless pods (Figure 1b), whereas F-wt plants showed normal flower and fruit development (Figure 1a). Observation of about 3400 St-M sterile plants showed that sterility was absolute with no seed development observed. Given that St-M plants could produce small pods, we speculated that the sterility of St-M was not caused by the pistil, but rather by abnormal pollen development.

**FIGURE 2:**
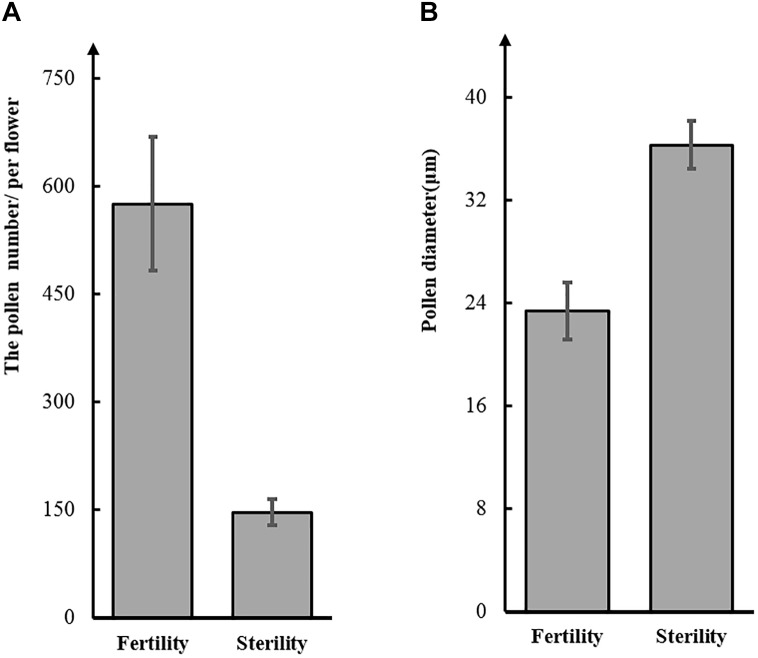
Number **(A)** and diameter **(B)** of pollen grains from the soybean sterile line (St-M) and fertile wild type (F-wt).

To test this hypothesis, we observed pollen grains microscopically. The pollen grains from St-M plants varied greatly in size and stained poorly with I_2_KI, whereas pollen grains from F-wt plants were uniform in size and were stained intensely with I_2_KI (Figure 1e,f). These observations indicated that the St-M pollen grains were aborted. Observation of pollens grains from St-M and F-wt with a SEM showed that F-wt produced small, rounded pollen grains full of cytoplasm, whereas St-M pollens grains were shriveled or collapsed (Figure 1c,d). In a previous study, cytological analysis suggested that male sterility in *ms1* soybean was caused by the failure of cytokinesis after telophase II of meiosis (Albertsen and Palmer, 1979). Lipids and starch were deposited in the enlarged but non-functional pollen grains (Albertsen and Palmer, 1979). To determine whether the mst-M gene was involved in similar process, the number and diameter of pollen grains in F-wt and St-M plants were evaluated. The results showed that the average total number of pollen grains per F-wt flower was about 580, whereas St-M flowers (150) had about one-quarter the pollen grains of F-wt (Figure 2A). The average diameter of St-M pollen grains was about 36 μm, which was approximately 1.6-fold greater than that of F-wt pollen grains (Figure 2B). We concluded that the sterility of St-M was caused by abortion of the pollen grains.

### Genetic Analysis of Sterility in St-M

The soybean cultivar JD12 was used as the male, female, or recurrent parent in crosses with St-M. The mode of sterility inheritance was determined as follows. In total, 32 pods developed from the cross St-M (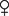) × JD12 (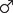), whereas no pods developed from the cross JD12 (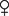) × St-M (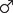) (Supplementary Table S1). This result implied that the pistil of St-M was normal and the sterility of St-M was caused by pollen abortion, which was consistent with the microscopic observations.

The inheritance of male sterility was evaluated in six progeny populations generated from the crosses St-M (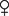) × JD12 (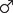) and JD12 (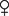) ×*F*_1_ [St-M (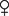) × JD12 (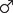)]. All *F*_1_ plants were fertile, and the *F*_2_ populations, which consisted of 418 plants, exhibited a good fit to the expected segregation ratio of 3:1 (fertility:sterility) (*p* = 0.82; Table 1). Progeny testing of the 316 *F*_2_ plants showed a 1:2:0 ratio of homozygous fertile:heterozygous fertile:homozygous sterile genotypes (*p* = 0.73). The segregation results for the *F*_1_, *F*_2_, and *F*_2:3_ populations suggested that the sterility of St-M was controlled by a single recessive gene. We designated the gene that controlled sterility “*male sterile-Mutant*” (*mst-M*).

**Table 1:** Inheritance analysis for sterility.

	Fertility	Seg	Sterility	Total	Expected ratio	χ^2^	*P*
S-M ( 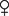 )			12	12			
*F*_1_	12			12			
*F_2_*	316		102	418	3:1	0.05	0.82
*F*_2:3_	112	204	0	316	1:2:0	0.63	0.73
JD12 ( 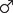 )	22			22			
BCF_1_(JD12 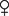 × F1 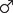 )	22			22			
BCF_1:2_	13	9		22	1:1	0.41	0.52
BCF_2_-Seg^∗^	378		109	487	3:1	1.64	0.20


*^∗^This population were comprised by the nine BCF_1:2_ plants with sterility segregation*.

Backcrosses were also performed to determine whether cytoplasm could affect sterility in the crosses. All 22 BCF_1_ progeny showed normal fertility like the female parent JD12 (Table 1). The 22 BCF_1_ progeny displayed a 1:1 ratio of homozygous fertile:heterozygous fertile genotypes (*p* = 0.52). The BCF_2_-Seg population, which consisted of 487 BCF_2_ individuals from nine segregated BCF_1:2_ lines, showed a good fit to a theoretical ratio of 3:1 (fertility:sterility) (*p* = 0.20). These results supported the conclusion that the sterility of St-M was controlled by a single recessive gene, and further implied that *mst-M* was not affected by the cytoplasm.

### Bulked Segregant Analysis Through Next-Generation Sequencing

**FIGURE 3:**
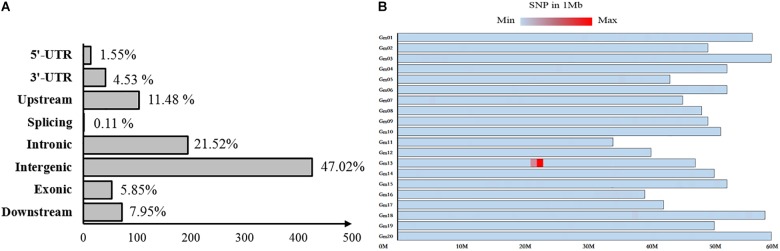
Distribution of variation between sterile and fertile DNA bulks. The distribution of single nucleotide polymorphisms in **(A)** coding and non-coding regions and **(B)** on individual chromosomes. The color shading represents SNP density per Mb on an individual chromosome.

Fertile and sterile DNA bulks, which were developed from a heterozygous fertile *F*_5:6_ line, were used for bulked segregant analysis to localize the *mst-M* gene to an individual chromosome through whole-genomic next-generation sequencing. A total of 906 variable SNPs of high quality (>100) were identified between the fertile and sterile bulks (Supplementary Table S2). More than 76% of the SNP variation was distributed in introns, intergenic regions, and downstream of coding regions, and was unlikely to cause *mst-M* function deficiency, whereas 1.5, 4.53, 11.48, 0.11, and 5.85% of the SNP variation was distributed in 5′-UTR, 3′-UTR, upstream, splicing, and exonic coding regions, respectively (Figure 3A). Almost all of the SNPs (95.92%) were distributed on chromosome 13 and 868 SNPs (95.81%) were distributed in the physical region of Chromosome 13. 21877872 to Chromosome 13. 22862641 (Figure 3B). These results strongly suggested that the *mst-M* gene was located on chromosome 13 approximately in the physical region of Chromosome 13. 21877872 to Chromosome 13. 22862641. All of the original re-sequencing data for the fertile and sterile DNA bulks have been submitted to the SRA database (SRA accession number PRJNA509511; BioSample accessions numbers SAMN10583719 and SAMN10583720, respectively).

### Genetic Mapping and Allele Analysis

**FIGURE 4:**
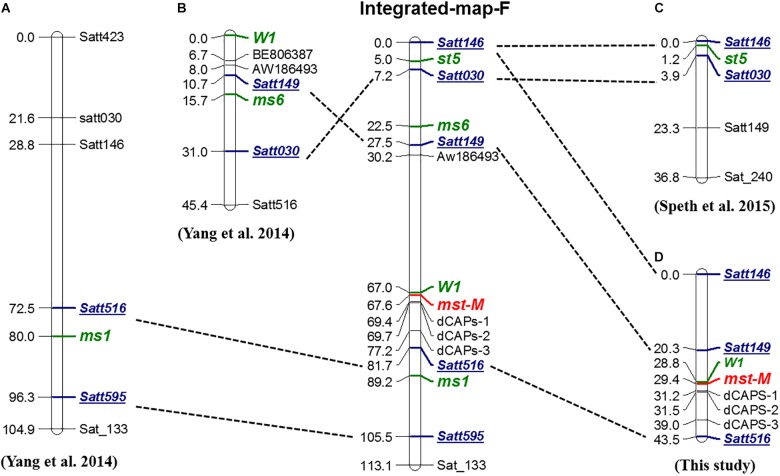
Genetic and integrated map of male-sterility genes in linkage group F. **(A–D)** Genetic maps for *ms1*, *ms6*, *st5*, and *mst-M*, respectively. The order of Integrated-map-F was based on republished consensus maps of Song et al. (2004). Green, bold, and italic fonts represent loci for identified genes. Blue, bold, and italic fonts with underlines are the consensus markers used for projection onto Integrated-map-F.

To determine the genetic location of the *mst-M* gene and the relationship of *mst-M* with three previously identified male-sterility genes (*ms1*, *ms6*, and *st5*) (Figure 4A–C), the marker distribution on chromosome 13 was analyzed. Three polymorphic SSR markers, three developed dCAPs markers, and a morphological marker (the flower color locus *W1*) were used to detect the genotypes of a large *F*_2_ population consisting of 1138 individual plants (Supplementary Table S3). Genetic mapping revealed that *mst-M* was flanked by *W1* and dCAPs-1. The genetic distance of the two markers was 0.6 and 1.8 cM from *mst-M* (Figure 4D). Five SSR markers, namely Satt146, Satt149, Satt030, Satt516, and Satt595, were used as consensus markers to project all four male-sterility genes on an integrated map of linkage group F. The integrated linkage map showed that the order of the consensus markers and sterility genes was as follows: Satt146 – (5.0 cM) – *st5* – (2.5 cM) – Satt030 – (15.3 cM) – *ms6* – (5.0 cM) – Satt149 – (39.5 cM) – *W1* – (0.6 cM) – *mst-M* – (14.1 cM) – Satt516 (7.5 cM) – *ms1* – (16.3 cM) – Satt595. These results suggested that *mst-M* was a newly identified male-sterility gene.

## Discussion

Two main kinds of male sterility have been well documented, CMS and GMS. For CMS, male sterility genes are usually maintained through crossbreeding with a maintainer line, and the CMS system has been widely used in rice hybrid production. However, although a three-line hybrid system based on CMS has been developed in soybean, large-scale hybrid seed production is difficult to achieve because of the extremely low frequency of natural cross-pollination. Therefore, the main limiting factor for CMS application in soybean hybrid seed production is the low frequency of natural cross-pollination. In the last 10 years, many researchers have tried to find external factors that can improve the frequency of natural cross-pollination. For example, insects such as alfalfa leaf-cutting bees and honey bees cannot only improve the soybean yield but also significantly enhance the outcrossing pod-set rate of CMS lines (Ortiz-Perez et al., 2008; Yang et al., 2008; Zhao et al., 2009; Wang et al., 2010; Milfont et al., 2013; Blettler et al., 2017; Dai et al., 2017), and a relatively low temperature before the flowering stage can also enhance the soybean outcrossing rate (Shimamura et al., 2010).

In contrast, GMS, in which the male sterility genes are mainly preserved in heterozygous plants, has been successful applied in the recurrent selection (RS) breeding system. The RS breeding system is an effective strategy that can accelerate the breeding process by pyramiding multiple elite genes. Although the need for emasculation and manual pollination limited the utility of RS in breeding processes for autogamous species, the introduction of male sterile genes to the RS breeding system changed the situation. In wheat, more than 40 new varieties have been developed based on the DMS wheat-based RS breeding platform, which uses the dominant dwarf gene *Rht-D1c* and male-sterile gene *Ms2* together (Liu and Yang, 1991; Ni et al., 2017; Xia et al., 2017). In soybean, RS breeding platform have been developed by using ms1 gene, and several commercial cultivars have also been developed using this RS breeding system, such as Jidou19, Jidou 20, and Jidou 21 (Zhao et al., 2010a,b, 2011). However, ms1 plant always produce a high frequency of twin seedlings which considerably disrupts the genetic diversity of RS breeding platform, and it is a difficult and time-consuming work to eliminate these useless twin seedlings. In contrast, the newly identified *mst-M* gene can cause absolute sterility plant which is very useful in maintaining the genetic diversity of RS breeding platform. More importantly, *mst-M* gene is closely linked to flower color and this linked morphological trait will facilitate breeders accelerating breeding process.

The exploitation of heterosis through the CMS system and the breeding of new varieties through the RS system are both economic, effective, and feasible approaches to improving crop yields. Male sterility plays an important role in the process of improving soybean yields. Unfortunately, although more than 20 genetic loci for male sterility have been identified in soybean, none of these loci have been developed for use in hybrid seed production. In comparison to research on maize or rice, there has been little fine-mapping and research on the molecular mechanism of male sterility in soybean, and none of the known male-sterility loci have been cloned using a genetic map-based approach. Therefore, newly identified male-sterility loci could provide alternative genetic material for developing hybrid seeds, and localization of the chromosomal positions of male-sterility genes will promote map-based cloning. In the present study, we identified and fine-mapped a novel male-sterility locus designated “*mst-M*”. Genetic analysis indicated that *mst-M* was closely linked with the flower color locus *W1*, and it was co-mapped to chromosome 13 with *ms1*, *ms6*, and *st5*. Because there is no sterile maintainer line for *ms1*, *ms6*, or *st5*, an allelism test of *mst-M* with *ms1*, *ms6*, or *st5* could not be performed. Therefore, the genetic locations and pollen morphology of *mst-M*, *ms1*, *ms6*, and *st5* are compared and discussed below.

The *ms1* gene was first reported in 1971, and a high frequency of twin seedlings was observed in male-sterile plants (Brim and Young, 1971; Kenworthy et al., 1973). Cytological analysis suggested that male sterility in *ms1* soybean was caused by the failure of cytokinesis after telophase II of meiosis (Albertsen and Palmer, 1979). However, lipid and starch deposits in the enlarged but non-functional pollen grains developed as in normal pollen grains (Albertsen and Palmer, 1979). In the present study, the pollen grains produced by *mst-M* male-sterile plants stained poorly with I_2_-KI (Figure 1f), which suggested that few or no starch grains were formed in the pollen grains. This result implied that the molecular mechanism of sterility caused by *mst-M* was different to that of *ms1*. An additional notable difference between *mst-M* and *ms1* male-sterile plants is the occurrence of twin seedlings; absolute sterility was observed in *mst-M* male-sterile plants and no twin seedlings were seen based on observation of thousands of individual plants. Thus, we speculate that *mst-M* is non-allelic to *ms1*.

Unlike *ms1*, the process of meiosis in *ms6* male-sterile plants is similar to that of fertile plants, with normal meiocytes forming in metaphase I and telophase II progressing normally but without microspore wall formation (Skorupska and Palmer, 1989). These observations imply that the pollen grains of *ms6* male-sterile plants will not vary greatly in size nor I_2_KI staining intensity, which differs markedly from the pollen grains of *mst-M* male-sterile plants (Figure 1d,f). Furthermore, a unique pleiotropic effect of the *ms6* allele is that it may cause a smaller flower size (Skorupska and Palmer, 1989), which differs from the phenotypic effects of the other male-sterility genes. These characteristics strongly suggest that *mst-M* is non-allelic to *ms6*.

Plants with the *st5* gene, which was identified as a desynaptic mutant gene, may show an absence of chromosome pairing at diakinesis (Palmer and Kaul, 1983). A notable characteristic of pollen grains in *st5* male-sterile plants is the wide variation in pollen grain size and poor staining with I_2_-KI (Palmer and Kaul, 1983). Both features are similar to those of pollen grains of *mst-M* male-sterile plants (Figure 1f). However, recombination percentages between *st5* and *W1* of about 26.7% were reported based on observation of two different *F*_2_ populations (Palmer and Kaul, 1983), and *st5* was mapped to the interval between the markers Satt146 and Satt030 with genetic distances of 5.0 and 2.5 cM, respectively (Speth et al., 2015). In the present investigation, male sterility was always accompanied by white flowers in the 1138 individual plants used for genetic mapping, and only six plants showed a chromosomal rearrangement between *mst-M* and *W1*. We localized *mst-M* to the interval between locus *W1* and the marker dCAPs-1 with genetic distances of 0.6 and 1.8 cM, respectively. Therefore, *mst-M* is distinctly non-allelic to *st5* based on the predicted physical positions for *mst-M* and *st5*.

The novel male-sterility gene identified in the present study provides an alternative genetic resource for development of a hybrid seed production system for soybean. Most importantly, the closely linked *W1* locus enables breeders to distinguish male-sterile plants at the seeding stage through hypostyle color. In addition, the present study lays a foundation for map-based cloning of male-sterility genes in soybean. Elucidation of the molecular mechanism of reproductive pathways would help to facilitate the cloning of male-sterility genes.

## Author Contributions

MZ and CY designed the experiments. QZ and YY analyzed the data. QZ, YT, and CY carried out the experiments. QZ and YY wrote the manuscript. MZ critically revised the manuscript.

## Conflict of Interest Statement

The authors declare that the research was conducted in the absence of any commercial or financial relationships that could be construed as a potential conflict of interest.

## Footnotes

1 http://helix.wustl.edu/dcaps/dcaps.html

2 https://www.soybase.org/

3 http://bio-bwa.sourceforge.net/

4 http://picard.sourceforge.net

## References

[B1] Adak M. S. Kibritci M. (2016). Effect of nitrogen and phosphorus levels on nodulation and yield components in faba bean (*Vicia faba* L.). *Legume Res.* 39 991–994.

[B2] Albertsen M. C. Palmer R. G. (1979). A comparative light-and electron-microscopic study of microsporogenesis in male sterile (ms,) and male fertile soybeans (*Glycine max* (L.) merr.). *Am. J. Bot.* 66 253–265. 10.1002/j.1537-2197.1979.tb06222.x

[B3] Bailey K. M. (2012). The research manual: design and statistics for applied linguistics. *Tesol Q.* 28 209–211.

[B4] Baumbach J. Rogers J. P. Slattery R. A. Narayanan N. N. Xu M. Palmer R. G. (2012). Segregation distortion in a region containing a male-sterility, female-sterility locus in soybean. *Plant Sci.* 195 151–156. 10.1016/j.plantsci.2012.07.003 22921009

[B5] Blettler D. C. Fagúndez G. A. Caviglia O. P. J. A. (2017). Contribution of honeybees to soybean yield. *Apidologie* 49 101–111. 10.1007/s13592-017-0532-4

[B6] Bohra A. Jha U. C. Adhimoolam P. Bisht D. Singh N. P. (2016). Cytoplasmic male sterility (CMS) in hybrid breeding in field crops. *Plant Cell Rep.* 35 967–993. 10.1007/s00299-016-1949-3 26905724

[B7] Brim C. A. Young M. F. (1971). Inheritance of a male-sterile character in soybeans. *Crop Sci.* 11 564–566. 10.2135/cropsci1971.0011183X001100040032x

[B8] Brown J. D. (2004). Questions and answers about language testing statistics: yates correction factor. *Shiken Jalt Testing and Evaluation Sig Newsletter* 8 22–27.

[B9] Cervantes-Martinez I. Sandhu D. Xu M. Ortiz-Perez E. Kato K. K. Horner H. T. (2009). The male sterility locus ms3 is present in a fertility controlling gene cluster in soybean. *J. Hered.* 100 565–570. 10.1093/jhered/esp054 19617521

[B10] Cervantes-Martinez I. Xu M. Zhang L. Huang Z. Kato K. K. Horner H. T. (2007). Molecular mapping of male-sterility loci *ms2* and *ms9* in Soybean. *Crop Sci.* 47 374–379. 10.2135/cropsci2006.03.0143

[B11] Chang Z. Chen Z. Wang N. Xie G. Lu J. Yan W. (2016). Construction of a male sterility system for hybrid rice breeding and seed production using a nuclear male sterility gene. *Proc. Natl. Acad. Sci. U.S.A.* 113 14145–14150. 10.1073/pnas.1613792113 27864513 PMC5150371

[B12] Chen L. Liu Y. G. (2014). Male sterility and fertility restoration in crops. *Annu. Rev. Plant Biol.* 65 579–606. 10.1146/annurev-arplant-050213-040119 24313845

[B13] Chen L. Y. Lei D. Y. Tang W. B. Xiao Y. H. (2011). Thoughts and practice of some problems about research and application of two-line hybrid rice. *Rice Sci.* 18 79–85. 10.1016/S1672-6308(11)60012-7

[B14] Cheng S. H. Zhuang J. Y. Fan Y. Y. Du J. H. Cao L. Y. (2007). Progress in research and development on hybrid rice: a super-domesticate in China. *Ann. Bot.* 100 959–966. 10.1093/aob/mcm121 17704538 PMC2759200

[B15] Dai J. Zhang R. Wei B. Nie Z. Xing G. Zhao T. (2017). Key biological factors related to outcrossing-productivity of cytoplasmic-nuclear male-sterile lines in soybean [*Glycine max* (L.) Merr.]. *Euphytica* 213:266 10.1007/s10681-017-2054-6

[B16] Delannay X. Palmer R. G. (1982). Genetics and cytology of the *ms4* male-sterile soybean. *J. Hered.* 73 219–223. 10.1093/oxfordjournals.jhered.a109621

[B17] Doyle J. (1991). DNA protocols for plants. *Mol. Tech. Taxon.* 57 283–293. 10.1007/978-3-642-83962-7_18

[B18] Fan Y. Yang J. Mathioni S. M. Yu J. Shen J. Yang X. (2016). PMS1T, producing phased small-interfering RNAs, regulates photoperiod-sensitive male sterility in rice. *Proc. Natl. Acad. Sci. U.S.A.* 113 15144–15149. 10.1073/pnas.1619159114 27965387 PMC5206514

[B19] Gao J. Chen M. Meng F. Yan Y. (2018). Effect of increasing panicle fertilizer on rice yield and nitrogen use efficiency. *J. Agric. Sci.* 43 1–4. 10.3389/fpls.2018.00999 30073007 PMC6060282

[B20] Graybosch R. A. Edge M. E. Delannay X. (1987). Somaclonal variation in soybean plants regenerated from the cotyledonary node tissue culture system 1. *Crop Sci.* 27 803–806. 10.2135/cropsci1987.0011183X002700040040x

[B21] Graybosch R. A. Palmer R. G. (1985). Male sterility in Soybean (*Glycine max*). II. Phenotypic Expression of the ms4 Mutant. *Am. J. Bot.* 72 1751–1764. 10.1002/j.1537-2197.1985.tb08448.x

[B22] Graybosch R. A. Palmer R. G. (1988). Male sterility in soybean-an overview. *Am. J. Bot.* 75 144–156. 10.1002/j.1537-2197.1988.tb12169.x

[B23] Hadley H. H. Starnes W. J. (1964). Sterility in Soybeans caused by Asynapsis. *Crop Sci.* 4 421–424. 10.2135/cropsci1964.0011183X000400040027x

[B24] Horner H. T. Palmer R. G. (1995). Mechanisms of genic male sterility. *Crop Sci.* 35 1527–1535. 10.2135/cropsci1995.0011183X003500060002x

[B25] Huang J. Z. Zhi-Guo E. Zhang H. L. Shu Q. Y. (2014). Workable male sterility systems for hybrid rice: genetics, biochemistry, molecular biology, and utilization. *Rice* 7:13. 10.1186/s12284-014-0013-6 26055995 PMC4883997

[B26] Jin W. Horner H. T. Palmer R. G. (1997). Genetics and cytology of a new genic male-sterile soybean [*Glycine max* (L.) Merr.]. *Sex Plant Rep.* 10 13–21. 10.1007/s004970050062

[B27] Jin W. Palmer R. G. Horner H. T. Shoemaker R. C. (1998). Molecular mapping of a male-sterile gene in Soybean. *Crop Sci.* 38 1681–1685. 10.2135/cropsci1998.0011183X003800060043x

[B28] Kenworthy W. J. Brim C. A. Wernsman E. A. (1973). Polyembryony in Soybeans. *Crop Sci.* 13 637–639. 10.2135/cropsci1973.0011183X001300060015x

[B29] Kosambi D. (2016). The estimation of map distances from recombination values. *Ann. Eugen.* 12 172–175. 10.1111/j.1469-1809.1943.tb02321.x

[B30] Liu B. Yang L. (1991). Breeding of dwarfing-sterile wheat and its potential values in wheat breeing. *Chin. Sci. Bull.* 36 1562–1562.

[B31] Liu C. Wang G. Gao J. Li C. Zhang Z. Yu T. (2018). Characterization, fine mapping and candidate gene analysis of novel, dominant, nuclear male-sterile gene *Ms53* in maize. *Euphytica* 214:52 10.1007/s10681-018-2132-4

[B32] Ma H. Sun R. Wu J. Zhang Y. (2018). Effect of controlled release urea and Zn/Mn urea on wheat yield and fertilizer utilization rate. *Chin. Agric. Sci. Bull.* 34 8–13.

[B33] Milfont M. D. O. Rocha E. E. M. Lima A. O. N. Freitas B. M. (2013). Higher soybean production using honeybee and wild pollinators, a sustainable alternative to pesticides and autopollination. *Environ. Chem. Lett.* 11 335–341. 10.1007/s10311-013-0412-8

[B34] Neff M. M. Turk E. Kalishman M. (2002). Web-based primer design for single nucleotide polymorphism analysis. *Trends Genet.* 18 613–615. 10.1016/S0168-9525(02)02820-2 12446140

[B35] Ni F. Qi J. Hao Q. Bo L. Luo M. C. Wang Y. (2017). Wheat Ms2 encodes for an orphan protein that confers male sterility in grass species. *Nat. Commun.* 8:15121. 10.1038/ncomms15121 28452349 PMC5414350

[B36] Ortiz-Perez E. Mian R. M. A. Cooper R. L. Mendiola T. Tew J. Horner H. T. (2008). Seed-set evaluation of four male-sterile, female-fertile soybean lines using alfalfa leafcutting bees and honey bees as pollinators. *J. Agric. Sci.* 146 461–469. 10.1017/S002185960700768X

[B37] Owen F. V. (1928). A sterile character in soybeans. *Plant Physiol.* 3 223–226. 10.1104/pp.3.2.223 16652565 PMC440003

[B38] Palmer R. G. (1974). A desynaptic mutant in soybean. *J. Hered.* 65 280–286. 10.1093/oxfordjournals.jhered.a108529 12369106

[B39] Palmer R. G. (2000). Genetics of four male-sterile, female-fertile soybean mutants. *Crop Sci.* 40 78–83. 10.1139/gen-2014-0018 24814801

[B40] Palmer R. G. Horner H. T. (2000). Genetics and cytology of a genic male-sterile, female-sterile mutant from a transposon-containing soybean population. *J. Hered.* 91 378–383. 10.1093/jhered/91.5.378 10994704

[B41] Palmer R. G. Kaul M. L. H. (1983). Genetics, cytology, and linkage of a desynaptic soybean mutants. *J. Hered.* 74 260–264. 10.1093/oxfordjournals.jhered.a109780

[B42] Palmer R. G. Sandhu D. Curran K. Bhattacharyya M. K. (2008). Molecular mapping of 36 soybean male-sterile, female-sterile mutants. *Theor. Appl. Genet.* 117 711–719. 10.1007/s00122-008-0812-5 18592206

[B43] Palmer R. G. Winger C. L. Albertsen M. C. (1978). Four independent mutations at the ms1, locus in Soybeans. *Crop Sci.* 18 727–729. 10.2135/cropsci1978.0011183X001800050008x

[B44] Ray J. D. Kilen T. C. Abel C. A. Paris R. L. (2003). Soybean natural cross-pollination rates under field conditions. *Environ. Biosafety Res.* 2 133–138. 10.1051/ebr:2003005 15612278

[B45] Rebeccaa S. Sarah P. Katie R. Brian T. Reidg P. Devinder S. (2011). Mapping eight male-sterile, female-sterile Soybean mutants. *Crop Sci.* 51 231–236. 10.2135/cropsci2010.06.0351

[B46] Shimamura S. Iimura K. Takamizo T. Ishimoto M. Hajika M. (2010). Effects of low temperature before flowering stage on natural out-crossing rates in soybean. *Japan. J. Crop Sci.* 79 316–321. 10.1626/jcs.79.316

[B47] Skorupska H. Palmer R. G. (1989). Genetics and cytology of the ms6 male-sterile Soybean. *J. Hered.* 80 304–310. 10.1093/oxfordjournals.jhered.a110858

[B48] Song Q. J. Marek L. F. Shoemaker R. C. Lark K. G. Concibido V. C. Delannay X. (2004). A new integrated genetic linkage map of the soybean. *Theor. Appl. Genet.* 109 122–128. 10.1007/s00122-004-1602-3 14991109

[B49] Speth B. Rogers J. P. Boonyoo N. VanMeter A. J. Baumbach J. Ott A. (2015). Molecular mapping of five soybean genes involved in male-sterility, female-sterility. *Genome* 58 143–149. 10.1139/gen-2015-0044 26213292

[B50] Voorrips R. E. (2002). Map chart: software for the graphical presentation of linkage maps and QTLs. *J. Hered.* 93 77–78. 10.1093/jhered/93.1.77 12011185

[B51] Wang D. Zhang L. Li J. Hu G. Wu Q. Jiang H. (2016). The restorer gene for soybean M-type cytoplasmic male sterility, Rf-m, is located in a PPR gene-rich region on chromosome 16. *Plant Breed.* 135 342–348. 10.1111/pbr.12357

[B52] Wang S. M. Wang Y. Q. Li J. P. Li M. H. Sun H. Zhao L. M. (2010). Seed production of soybean cytoplasmic male sterile (CMS) line under field conditions. *Soyb. Sci.* 29 385–389.

[B53] Wang Z. Li J. Chen S. Heng Y. Chen Z. Yang J. (2017). Poaceae-specific MS1 encodes a phospholipid-binding protein for male fertility in bread wheat. *Proc. Natl. Acad. Sci. U.S.A.* 114 12614–12619. 10.1073/pnas.1715570114 29109252 PMC5703327

[B54] Wen Z. Shen J. Martin B. Li H. Zhao B. Yuan H. (2016). Combined applications of nitrogen and phosphorus fertilizers with manure increase maize yield and nutrient uptake via stimulating root growth in a long-term experiment. *Pedosphere* 26 62–73. 10.1016/S1002-0160(15)60023-6

[B55] Willingham M. C. Rutherford A. V. (1984). The use of osmium-thiocarbohydrazide-osmium (OTO) and ferrocyanide-reduced osmium methods to enhance membrane contrast and preservation in cultured cells. *J. Histochem. Cytochem.* 32 455–460. 10.1177/32.4.6323574 6323574

[B56] Xia C. Zhang L. Zou C. Gu Y. Duan J. Zhao G. (2017). A TRIM insertion in the promoter of Ms2 causes male sterility in wheat. *Nat. Commun.* 8:15407. 10.1038/ncomms15407 28497807 PMC5437302

[B57] Xie K. Wu S. Li Z. Zhou Y. Zhang D. Dong Z. (2018). Map-based cloning and characterization of Zea mays male sterility33 (*ZmMs33*) gene, encoding a glycerol-3-phosphate acyltransferase. *Theor. Appl. Genet.* 131 1363–1378. 10.1007/s00122-018-3083-9 29546443 PMC5945757

[B58] Xu X. Zhang S. Liang K. (2007). Progress and discussion in breeding of Indica Rice CMS lines in China. *Chin. Agric. Sci. Bull.* 32 176–180.

[B59] Yang G. H. Li J. P. Li M. H. Liu J. W. (2008). Effect of using the male of alfalfa leaf-cutting bee, megachile rotundata, on pollination of Soybean CMS Lines in cages. *J. Jilin Agric. Sci.* 33 11–13.

[B60] Yang S. P. Duan M. P. Meng Q. C. Qiu J. Fan J. M. Zhao T. J. (2010). Inheritance and gene tagging of male fertility restoration of cytoplasmic-nuclear male-sterile line NJCMS1A in soybean. *Plant Breed.* 126 302–305. 10.1111/j.1439-0523.2007.01356.x

[B61] Yang Y. Speth B. D. Boonyoo N. Baumert E. Atkinson T. R. Palmer R. G. (2014). Molecular mapping of three male-sterile, female-fertile mutants and generation of a comprehensive map of all known male sterility genes in soybean. *Genome* 57 155–160. 10.1139/gen-2014-0018 24814801

[B62] Zhang Y. Li Y. Zhang J. Shen F. Huang Y. Wu Z. (2008). Characterization and mapping of a new male sterility mutant of anther advanced dehiscence (t) in rice. *J. Genet. Genomics* 35 177–182. 10.1016/S1673-8527(08)60024-7 18355761

[B63] Zhao L. M. Sun H. Peng B. Li J. P. Wang S. M. Li M. H. (2009). Pollinator effects on genotypically distinct soybean cytoplasmic male sterile lines. *Crop Sci.* 49 2080–2086. 10.2135/cropsci2008.11.0662

[B64] Zhao N. Xu X. Wamboldt Y. Mackenzie S. A. Yang X. Hu Z. (2016). MutS HOMOLOG1 silencing mediates ORF220 substoichiometric shifting and causes male sterility in *Brassica juncea*. *J. Exp. Bot.* 67 435–444. 10.1093/jxb/erv480 26516127 PMC4682445

[B65] Zhao S. Liu B. Yang C. Zhang M. (2011). Breeding and cultivation points of new high protein soybean variety jidou 21. *Agric. Sci. Tech. Commun.* 6 184–185.

[B66] Zhao S. Zhang M. Yang C. Jiang C. Liu B. (2010a). Breeding and cultivation points of a new high oil soybean variety jidou 19. *Crop J.* 629:128.

[B67] Zhao S. Zhang M. Yang C. Jiang C. Liu B. (2010b). Breeding and cultivation points of a new high protein soybean variety jidou 20. *Agric. Sci. Tech. Commun.* 1 145–146.

[B68] Zou T. Liu M. Xiao Q. Wang T. Chen D. Luo T. (2018). OsPKS2 is required for rice male fertility by participating in pollen wall formation. *Plant Cell Rep.* 37 759–773. 10.1007/s00299-018-2265-x 29411094

