# Supplementary material for: Identification and Mapping of a New Soybean Male-Sterile Gene, mst-M
Source: Front Plant Sci. 2019 Feb 6;10:94. doi: 10.3389/fpls.2019.00094 (PMC6372514; doi:10.3389/fpls.2019.00094)
Supplement: Supplementary file 1 [file Table_1.DOC]

**Table S1 Crosses made in this study**

|  | S-M（♂） | JD12（♂） | F_1_ (♂） |
| --- | --- | --- | --- |
| S-M（♀） | - | 32/252 | - |
| JD12（♀） | 0/339 | - | 22/243 |
