# Supplementary material for: Identification and Mapping of a New Soybean Male-Sterile Gene, mst-M
Source: Front Plant Sci. 2019 Feb 6;10:94. doi: 10.3389/fpls.2019.00094 (PMC6372514; doi:10.3389/fpls.2019.00094)
Supplement: Supplementary file 3 [file Table_3.DOC]

**Table S3 The information of primers used in this study**

| **Marker** | **Physical location** | | **Primer** | **Restriction**  **enzyme** | **Product**  **Size** |
| --- | --- | --- | --- | --- | --- |
|  | **Wm82.a1.v1** | **Wm82.a2.v1** |  |  |  |
| Satt516 | 20933787-20933843 | 22489376-22489620 | **F:** GCGTTAGCACTATTTTTTTACAAGA; **R:**GCGCCGTTCCTCTTTACTTTAT | - | 253 bp |
| Satt146 | 13078671-13078718 | 1357480-1357766 | **F:**AAGGGATCCCTCAACTGACTG; **R:**GTGGTGGTGGTGAAAACTATTAGAA | - | 287 bp |
| Satt149 | 4976853-4976900 | 16855019-16855292 | **F:**TTGCACATTCTTTTTGGTAAACAGTCATAA **R:**GTTGGAGGCCATAGTCACATTAATCTTAGA | - | 274 bp |
| dCAPS-1 | - | 21931375 | **F:**TAAAGCAATATCTTCCCCAAAACAAAAC**C**C **R:**GATGGGAAACTGAATCATAG | *SmaI* | 172 bp |
| dCAPS-2 | - | 21948774 | **F:**ATGCCTTTGATGCTTTTGTC **R:**AGAATAATTCCAAGAAAACACGTGTT**A**T | *VspI* | 146 bp |
| dCAPS-3 | - | 22254953 | **F:**AGCTTTGCCTTTTTCTTCAG **R:**GAATCTGAGGATGAGTATCCCATTCC**G**AT | *DpnI* | 255 bp |

^#^The underlined bold font indicates the position of introduced SNP variation.
